# Patient Survival in Renal Allograft Failure: A Time-dependent Analysis

**DOI:** 10.5812/numonthly.13589

**Published:** 2013-10-30

**Authors:** Moghaddameh Mirzaee, Jalal Azmandian, Hojjat Zeraati, Mahmood Mahmoodi, Kazem Mohammad, Faramarz Fazeli, Mohammad-Reza Ebadzadeh

**Affiliations:** 1 Department of Epidemiology and Biostatistics, Tehran University of Medical Sciences, Tehran, IR Iran; 2 Physiology Research Center, Departments of Nephrology, Urology and Renal Transplantation, Kerman University of Medical Sciences, Kerman, IR Iran; 3 Departments of Nephrology, Urology and Renal Transplantation, Kerman University of Medical Sciences, Kerman, IR Iran; 4 Department of Urology, Zahedan University of Medical Sciences, Zahedan, IR Iran

**Keywords:** Transplantation, Homologous, Kidney Transplantation, Survival

## Abstract

**Background:**

To improve patient survival after a renal transplant, it is important to detect which variables affect it.

**Objectives:**

This study aimed to assess the effect of renal allograft failure on patient survival.

**Patients and Methods:**

This retrospective cohort study included 405 renal transplant patients from Kerman University of Medical Sciences hospital, Kerman, Iran from 2004 to 2010. Kaplan-Meier method was used to estimate survival rates of patients, and time-dependent Cox regression was used to examine the effect of allograft failure on patient survival.

**Results:**

During 4.06 years (median) of follow-up 28 (6.9%) patients died and 20 (71.4%) of dead patients had allograft failure. Survival rate of patients with allograft failure at 1-, 3-, 5-, and 7-year were 0.98, 0.8, 0.53, and 0.53, respectively; in patients with allograft function these values were 0.99, 0.98, 0.97, and 0.96, respectively. The unadjusted death rate was 0.5 per 100 patient years for the maintained allograft function, which increased to 9 per 100 patient years for patients following allograft failure. In fully adjusted model the risk of death increased in patients with allograft failure (HR = 2.09; 95% CI: 1.56-2.81), pretransplant diabetes (HR = 2.81; 95% CI: 1.2-6.7), patients with BMI ≥ 25 (vs. 18.5 ≤ BMI < 25) (HR = 3.56; 95% CI: 1.09-11.6). With an increase in recipient age this risk increased (HR = 1.04 per year increase; 95% CI: 1.01-6.7). Receiving a living kidney transplant decreased this risk (HR = 0.52; 95% CI: 0.39-0.69).

**Conclusions:**

An increase in recipient age and BMI, affliction with diabetes, allograft failure, and receiving deceased kidney transplant increased the risk of death.

## 1. Background

Each year the number of patients with end-stage renal disease (ESRD) increases at a rate of 7-8% (1). The highest prevalence rates of ESRD were reported in Taiwan and Japan with 2447 and 2205 cases per million population in 2009 (2). The rate was 467 per million in Iran in 2006 (3). These patients usually choose dialysis or renal transplant (RT). Most of them choose RT since it improves the quality of life and is cost-effective compared to dialysis (1, 4, 5). Canada has the first rank of RT in the world (2), and Iran has the first rank of RT in the Middle East and forth rank in the world (5). Despite the decrease in mortality of patients with ESRD after RT, their survival remains less than the general population (6). In addition to patient mortality, allograft failure (AF) is a major concern in RT patients. Despite improvements in the survival of renal allograft, many patients still experience progressive AF (7) as one- and five-year allograft survival rates in deceased donor have been reported to be 0.91 and 69.3, and in living donor 95.6 and 81.9 (8). In Iran, one- and five-year allograft survival rates are 0.82 and 0.63 in general (9). Some studies in the United States and Canada have shown that mortality rate in patients with renal AF was over three times higher than those who maintained allograft function (10, 11). In addition, some studies have shown only high mortality rates in patients with renal AF but they have not compared this rate with patients who maintained allograft function (12, 13). In spite of these findings, it is suggested to repeat such observational studies in other populations due to differences between health care systems and patient characteristics. In addition, to date no studies have evaluated patient survival of renal transplants in Southeastern Iran.

## 2. Objectives

Therefore, the aim of this study was to determine the effect of renal AF on patient survival after adjusting for confounding variables in a recent cohort of kidney transplant recipients. Time-dependent Cox regression was used to model the effect of renal AF on patient survival, and Kaplan-Meier estimators were used to estimate one-, three-, five- and seven-year patient survival rates. 

## 3. Patients and Methods

### 3.1. Study Subjects and Design

This retrospective cohort study was performed on 405 patients with ESRD, who had chosen RT therapy in Kerman University of Medical Sciences hospital, Kerman, Iran from 2004 to 2010. Patients undergoing repeated transplants were excluded from the study. The patients were followed from the date of RT until death or 2011. The patients were censored if they were alive at the end of the study or dead by any cause other than RT such as accident, stroke or cardiovascular disease. The most common cause of death was infection and malignancy.

### 3.2. Potential Confounders 

Clinical and demographic information was collected from patient records in the hospital and follow-up was performed by the nephrology clinic. Donors’ data included age, gender and blood type. Information on recipients were age, gender, education (illiterate, up to high school, high school and upper), marital status (single or married; single means not married at all, and married means married at least for once), body mass index (BMI) (< 18.5, 18.5-25, ≥ 25), donor type (deceased, living), ABO matching (donors and recipients matched or not matched in blood type and Rh factor), pretransplant variables such as hypertension, diabetes, and duration of time on dialysis therapy [months] (≤ 6, 6-24, ≥ 24). Post-transplant variables included allograft status (allograft failed, not failed).

### 3.3. Statistical Analysis

Baseline characteristics were described by using mean values ± standard deviations for continuous and frequency (percentages) for categorical data. To compare baseline characteristics between those with and without renal AF, chi-squared test was used for categorical variables and t-test for continuous variables. In multivariate analysis time-dependent Cox regression was used because the value of renal AF can change at any time point after transplant. The effect of the variable donor type was not constant at time intervals; as a result, these variables were treated as time-dependent variables. Therefore, logarithm and identity functions of survival time were used for multiplying by these variables. Finally, one of these two functions was chosen by using the Akaike information criterion (AIC), which assesses the goodness of fit of statistical model (14). A lower value of AIC suggests a better model. Significant variables on patient survival were detected by using a step-wise method. Patient survival rates at 1-, 3-, 5- and 7-year intervals were estimated by Kaplan-Meier method. All the statistical analyses were performed using Stata 8. A two-sided P < 0.05 was considered statistically significant.

## 4. Results

### 4.1. Baseline Characteristics

A total of 405 RT patients undergone transplantation between 2004 and 2010 were included in this study. During the 4.06-year (median) follow-up, 28 patients (6.9%) died and 51 (12.6%) patients had AF, leading to the death of 20 (39.21%) patients. 

Table 1 lists baseline characteristics of recipients and donors for patients with and without AF. According to this table patients with AF were more likely to have received deceased kidney transplants, having pretransplant hypertension, and pretransplant diabetes compared to patients who maintained allograft function. The distributions of other variables, such as donor characteristics (age, gender) and recipient characteristics (age, gender, BMI, etc.), were the same between the two groups. 

**Table 1.** Baseline Characteristics of Patients With Renal Transplant^a ^

Characteristics	Allograft Failed	No Allograft Failed	Total	P Value
**Patients, No. (%)**	51 (12.6)	354 (87.4)	405 (100)	-
**Age, mean ± SD, y**	37 ± 16	38 ± 15	38 ± 15	0.77
**Gender, No. (%)**				0.45
Female	25 (49)	154 (43.5)	179 (44.2)	
Male	26 (51)	200 (56.5)	226 (55.8)	
**Education, No. (%)**				0.74
Illiterate	12 (23.5)	69 (19.5)	81 (20)	
Up to high school	29 (56.9)	203 (57.3)	232 (57.3)	
High school and upper	10 (19.6)	82 (23.2)	92 (22.7)	
**Body Mass Index (BMI), No. (%), kg/m^2^**				0.42
≤ 18.5	25 (49)	144 (40.7)	169 (41.7)	
18.5-25	18 (35.3)	130 (36.7)	148 (36.5)	
≥ 25	8 (15.7)	80 (22.6)	88 (21.7)	
**Donor type, No. (%)**				0.06
Deceased	11 (21.6)	43 (12.1)	54 (13.3)	
Living	40 (78.4)	311 (87.9)	351 (86.7)	
**Pretransplant hypertension, No. (%)**				0.001
No	25 (49)	254 (71.8)	279 (68.9)	
Yes	26 (51)	100 (28.2)	126 (31.1)	
**Pretransplant diabetes, No. (%)**				0.004
No	36 (70.6)	305 (86.2)	341 (84.2)	
Yes	15 (29.4)	49 (13.8)	64 (15.8)	
**Pretransplant dialysis time, No. (%), mo **				0.4
≤ 6	18 (35.3)	148 (41.8)	166 (41)	
6-24	18 (35.3)	129 (36.4)	147 (36.3)	
≥ 24	15 (29.4)	77 (21.8)	92 (22.7)	
**ABO matching, No. (%)**				0.32
No	4 (8.3)	45 (13.4)	49 (12.7)	
Yes	44 (91.7)	292 (86.6)	336 (87.3)	
**Donor age, mean ± SD, y**	28 ± 6	29.09 ± 7.38	28.08 ± 7.2	0.93
**Donor gender, No. (%)**				0.08
Female	19 (37.3)	91 (25.7)	110 (27.2)	
Male	32 (62.7)	263 (74.3)	295 (72.8)	

^a^ Values expressed as Mean ± standard deviation for continues or number (percent) for categorical variables.

**Figure 1.**
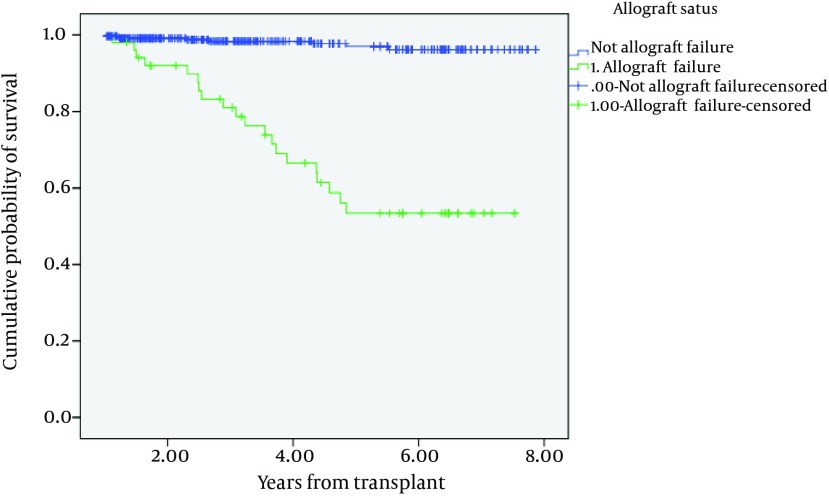
Kaplan-Meier of Cumulative Probability Survival of Patient According to Renal Allograft Status

Figure 1 shows the cumulative probability of surviving patients with and without AF. Kidney recipients with AF were significantly more probable to experience death during the follow-up period. 

Survival rates after 1-, 3-, 5- and 7- year for patients with AF were 0.98, 0.8, 0.53, and 0.53, respectively, and for patients who maintained allograft function the rates were 0.99, 0.98, 0.97 and 0.96, respectively (Table 2). 

The unadjusted death rate was 0.5 per 100 patient years for those who maintained allograft function, which increased to 9 per 100 patient years following AF (Table 3). 

**Table 2.** Kaplan-Meier Survival and 95% CI According to Renal Allograft Failure

Survival Times	Patient Survival (95%CI^a^)		
	Allograft Failure	No Allograft Failure	All Patients
**1 year**	0.98 (0.86-0. 99)	0.99 (0.97-0.99)	0.99 (0.98-0.99)
**3 year**	0.8 (0.66-0.89)	0.98 (0.96-0.99)	0.95 (0.92-0.97)
**5 year**	0.53 (0.37-0.67)	0.97 (0.93-0.98)	0.89 (0.84-0.92)

^a^ Abbreviation: CI, confidence interval.

**Table 3.** Unadjusted Death Rates According to Renal Allograft Failure

Renal Allograft	Person-years	Death	Rates	95%CI
**Allograft failure**	220.71	20	0.09	0.006-0.14
**No allograft failure**	1441.05	8	0.005	0.002-0.011
**Total**	1661.75	28	0.02	0.011-0.024

### 4.2. Multivariate Analysis

By using time-dependent Cox regression, a significant association was noted between renal AF and patient survival (Table 4). After controlling for donor type, pretransplant diabetes, recipient age and BMI, the relative risk of death was two times higher for patients with AF in comparison to those who maintained allograft function (adjusted hazard ratio of 2.09; 95% CI: 1.56-2.81; P < 0.0001) (Table 4). 

**Table 4.** Multivariate Time-dependent Cox Regression Analysis for Time to Death of Renal Transplant Patients

Variable	Adjusted Hazard Ratio	95%CI	P Value
**Renal allograft status^a^**			
No allograft failure	1		
Allograft failure	2.09	(1.56-2.81)	< 0.0001
**Donor type^a^**			
Deceased	1		
Living	0.52	(0.39-0.69)	< 0.0001
**Diabetes**			
No	1		
Yes	2.81	(1.2-6.7)	0.02
**BMI, kg/m^2^**			
< 18.5	2.57	(0.92-7.2)	0.07
18.5-25	1		
≥ 25	3.56	(1.09-11.6)	0.03
**Age recipient, y**	1.04	(1.01-1.08)	0.01

^a^ Analyzed as a Time –dependent variable.

AF was considered a time-dependent variable because its value changed over time. Increased recipient age (adjusted hazard ratio of 1.04 per year increase; 95% CI: 1.01-1.08; P = 0.01) and affliction with diabetes (adjusted hazard ratio of 2.81; 95% CI: 1.2-6.7; P = 0.02) increased the risk of death. Receiving a living kidney transplant reduced the risk of death by 48% compared to receiving a deceased kidney transplant (adjusted hazard ratio of 0.52; 95% CI: 0.39-0.69; P < 0.0001) (Table 4). Donor type was considered a time-dependent variable because its effect on patient survival changed over time. However, the risk of death was higher in patients with (BMI ≥ 25) compared to patients with normal weight (18.5 ≤ BMI < 25) (adjusted hazard ratio of 3.56; 95% CI: 1.09-11.6; P = 0.03) but the risk of death was the same for patients who were underweight (BMI < 18.5) compared to patients with normal weight (adjusted hazard ratio of 2.57; 95% CI: 0.92-7.2; P = 0.07) (Table 4). 

The above results were obtained by considering identity function of survival time for multiplying by time-dependent variables (AIC = 237.46) because this fit was better than considering logarithm survival time as a function of time for multiplying by time-dependent variables (AIC = 248.6).

## 5. Discussion

The results of this study showed that 12.6% of patients had AF after RT, with the death rate of 39.2% for them during the 4.06-year median follow-up period; it was also shown that that unadjusted rate of death was higher in patients with AF compared to patients with allograft function.

In addition, it was shown that renal AF had a significant association with patient survival. In this context, the risk of death was two folds in patients with renal AF compared to those who maintained allograft function after controlling important factors.

The results of this study are almost consistent with those of other studies. The US Renal Data System reported that one quarter of patients with renal allograft transplant had AF after 5 years. Therefore, it was reported that the five-year survival rate of renal allograft in deceased kidney transplant was 75% (15); the rate of AF after 7 years in all the patients (living and deceased kidney transplant) in this study was 12.6%. In this study the follow-up period was longer and all the patients (not merely deceased kidney transplant patients) were considered in the analysis, resulting in the difference between 12.6% and 25%. 

In addition to AF, it is necessary to consider survival of these patients. Some studies such as a study that used the United States renal data system (USRDS) concentrated only on the unadjusted rate of mortality of patients in renal allograft transplants and it was reported that mortality rate in patients who maintained allograft function was 2.81 per 100 patient years, which increased to 9.42 per 100 patient years for patients following AF; therefore, the unadjusted risk of death was nearly three folds in renal allograft patients (10, 12, 16). However, the results of this study showed that the unadjusted rate of mortality in patients with AF was 18 folds higher. The difference between the two studies might be attributed to the fact that in the present study the characteristics of dead patients with AF and those who maintained allograft function were different but after adjusting these characteristics, the differences decreased. The results of this study, after controlling important factors, showed that the risk of death in patients with AF was two folds compared to patients who preserved allograft function. This result was consistent with that of other studies showing adjusted risk of death in patients with renal AF. Knoll et al. showed that renal AF increased the risk of death by over three folds compared to patients who maintained transplant function (11).

Some studies have considered the association between renal AF and patient survival indirectly, showing that serum creatinine was strongly associated with mortality one year after RT (17). Meier-Kriesche et al. by using USRD showed that a serum creatinine level of 1.5-1.6 mg/dL at 1 year increased the risk of cardiovascular death 1.19 times (17). However, no serial clinical measurements, such as proteinuria, were available in the present study to detect such an association; therefore, in the present study only the association between complete loss of renal function and patient survival was considered. 

Today, the number of patients waiting for deceased kidney transplants is increasing. However, the results of this study showed that recipients who received a living kidney transplant had better survival in comparison with those who received a deceased kidney transplant, consistent with the results of other studies (11, 18). In contrast, a study in Iran has shown that 1- and 2-year survival rates were the same between living and deceased kidney transplants (19). The difference might be attributed to the follow-up period; in this study the median follow-up period was 4.06 years. Recipient age is known as an important factor for patient survival; the risk of death increases with advancing age. Noppakun et al. showed that the risk of death increased with an increase in recipient age with living kidney transplants (20).

In this study the effect of pretransplant diabetes on patient survival was evaluated and it was shown to be a significant variable. The results of this study showed that pretransplant diabetes decreased the survival rate. In addition, Knoll et al. showed in Canada that pretransplant diabetes decreased patient survival (11). In contrast, a study performed in Iran between March 2007 and September 2009 showed that patients with diabetes had the same survival rate as nondiabetic patients (21). The difference might be attributed to the fact that some patients developed diabetes after the transplant and in the present study only pretransplant diabetes was considered. 

In this study obesity was known as a risk factor of death in RT patients. Some studies have aimed to evaluate the impact of obesity on patient survival in renal allograft. Ellen et al. reported that obese patients run 20-40% higher risk for death in renal allograft (22). Yamamoto et al. showed that 1- and 3-year survival rates of RT were the same between obese and nonobese patients, but 5- and 7-year survival rates were different between the two groups (23).

This study had some limitations. First, no information was available about matching factors between donors and recipients except for blood type. It is possible that mismatching between donors and recipients was associated with patient survival. Second, there was no access to laboratory data except for serum creatinine at the time of AF to explain why these patients ran a higher risk of death.

In conclusion, this study showed that renal AF is a significant predictor of patient survival with RT in Iranian population. In fact, patient survival rate can be increased by assessing factors affecting renal AF in future.
